# Poly[aqua­(μ_2_-pyrimidine-2-carboxyl­ato-κ^4^ *O*,*N*:*O*′,*N*′)(nitrato-κ*O*)cadmium]

**DOI:** 10.1107/S1600536812041645

**Published:** 2012-10-13

**Authors:** Orrasa In-noi, Kittipnog Chainok, David J. Harding

**Affiliations:** a Department of Chemistry, Faculty of Science, Ubon Ratchathani Ratjabhat University, Muang, Ubon Ratchathani 34000, Thailand; b Department of Chemistry, Faculty of Science, Naresuan University, Muang, Phitsanulok 65000, Thailand; c Molecular Technology Research Unit, Department of Chemistry, Walailak University, Nakhon Si Thammarat 80161, Thailand

## Abstract

In the title polymer, [Cd(C_5_H_3_N_2_O_2_)(NO_3_)(H_2_O)]_*n*_, the Cd^II^ atom is seven-coordinate in a distorted capped octa­hedral geometry by two N atoms of two different pyrimidine dicarboxyl­ate (pmc) ligands, three O atoms from three separate pmc ligands, and two O atoms of disordered nitrate anions or water mol­ecules. The Cd^II^ atoms are bridged by the pmc ligands in a chelating/bridging bis-bidentate and chelating bidentate mode, forming sheets parallel to (20-1). The sheets are further linked into a three-dimensional supra­molecular network *via* classical O—H⋯O hydrogen bonds involving the nitrate anions and coordinating water mol­ecules. Intra­molecular O—H⋯O hydrogen bonding is also observed. The non-coordinating nitrate O atoms are disordered over two sets of sites with occupancies of 0.57 (7) and 0.43 (7).

## Related literature

For the synthesis, structures and properties of related cadmium coordination polymers with the pyrimidine dicarboxyl­ate ligand, see: Sava *et al.* (2008 ▶); Zhang *et al.* (2008 ▶); Rodríguez-Diéguez *et al.* (2007 ▶). For π–π inter­actions, see: Janiak (2000 ▶).
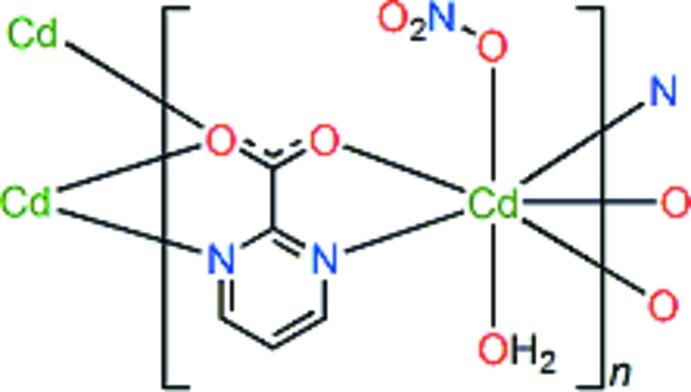


## Experimental

### 

#### Crystal data

[Cd(C_5_H_3_N_2_O_2_)(NO_3_)(H_2_O)]
*M* *_r_* = 315.52
Monoclinic, 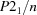

*a* = 8.1963 (2) Å
*b* = 10.1554 (3) Å
*c* = 11.0057 (3) Å
β = 107.435 (3)°
*V* = 873.99 (4) Å^3^
*Z* = 4
Mo *K*α radiation
μ = 2.52 mm^−1^
*T* = 298 K
0.23 × 0.20 × 0.14 mm

#### Data collection

Bruker SMART APEX CCD area detector diffractometer
Absorption correction: multi-scan (*SADABS*; Bruker, 2001 ▶) *T*_min_ = 0.596, *T*_max_ = 0.720
5450 measured reflections
2030 independent reflections
1780 reflections with *I* > 2σ(*I*)
*R*_int_ = 0.031

#### Refinement

*R*[*F* ^2^ > 2σ(*F* ^2^)] = 0.028
*wR*(*F* ^2^) = 0.070
*S* = 1.04
2030 reflections
163 parameters
56 restraints
H atoms treated by a mixture of independent and constrained refinement
Δρ_max_ = 1.52 e Å^−3^
Δρ_min_ = −0.64 e Å^−3^

### 

Data collection: *SMART* (Bruker, 2001 ▶); cell refinement: *SAINT* (Bruker, 2002 ▶); data reduction: *SAINT*; program(s) used to solve structure: *SHELXS97* (Sheldrick, 2008 ▶); program(s) used to refine structure: *SHELXL97* (Sheldrick, 2008 ▶); molecular graphics: *ORTEP-3 for Windows* (Farrugia, 1997 ▶) and *DIAMOND* (Brandenburg, 2006 ▶); software used to prepare material for publication: *publCIF* (Westrip, 2010 ▶).

## Supplementary Material

Crystal structure: contains datablock(s) global, I. DOI: 10.1107/S1600536812041645/tk5156sup1.cif

Supplementary material file. DOI: 10.1107/S1600536812041645/tk5156Isup2.cdx

Structure factors: contains datablock(s) I. DOI: 10.1107/S1600536812041645/tk5156Isup3.hkl

Additional supplementary materials:  crystallographic information; 3D view; checkCIF report

## Figures and Tables

**Table 1:** Selected bond lengths (Å)

Cd1—N1	2.376 (3)
Cd1—N2^i^	2.353 (3)
Cd1—O1	2.463 (2)
Cd1—O1^ii^	2.371 (2)
Cd1—O2^i^	2.411 (2)
Cd1—O3	2.382 (3)
Cd1—O4	2.339 (2)

Symmetry codes: (i) 
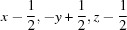
; (ii) 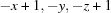.

**Table 2:** Hydrogen-bond geometry (Å, °)

*D*—H⋯*A*	*D*—H	H⋯*A*	*D*⋯*A*	*D*—H⋯*A*
O3—H3*A*⋯O4^ii^	0.90 (1)	1.98 (1)	2.871 (4)	173 (5)
O3—H3*B*⋯O5*A* ^iii^	0.90 (1)	2.17 (2)	3.045 (14)	164 (6)
O3—H3*B*⋯O5*B* ^iii^	0.90 (1)	2.04 (3)	2.876 (13)	154 (6)

Symmetry codes: (ii) 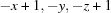; (iii) 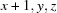.

**Fig. 1.**
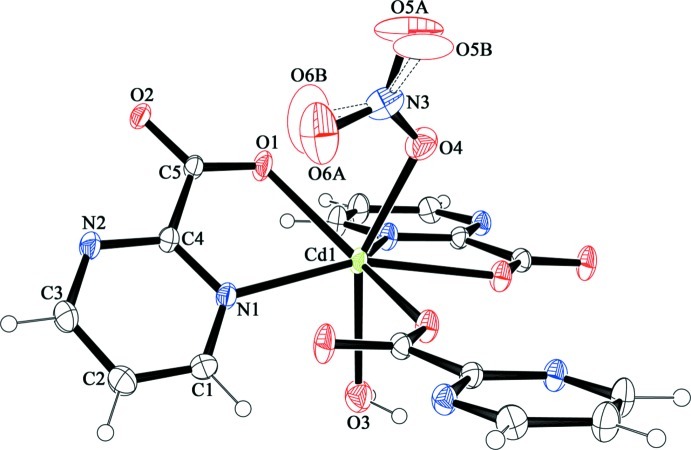
Displacement ellipsoid plot at the 35% probability level of the immediate coordination geometry about the cadmium(II) centre in I. The asymmetric unit is labelled.

**Fig. 2.**
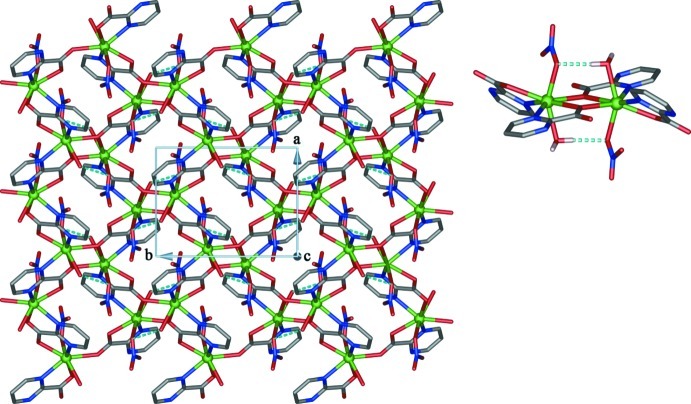
View of the two dimensional sheets parallel to (201) in I, showing inversion-related pairs of intramolecular O—H···O hydrogen bonds (dashed lines).

**Fig. 3.**
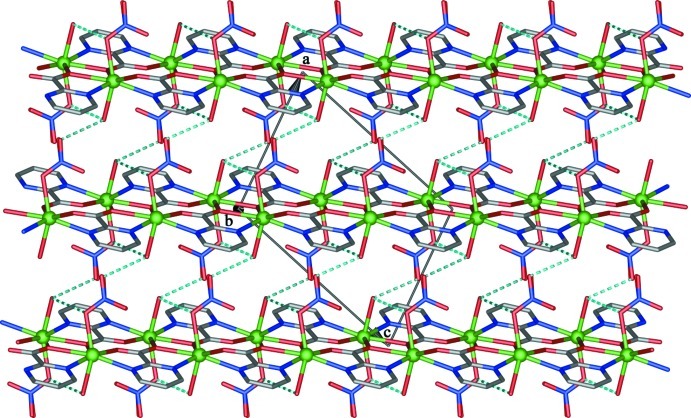
View of the three-dimensional supramolecular network showing the intermolecular O—H···O hydrogen bonds (dashed lines) between adjacent layered sheets of I.

## supplementary crystallographic information

### Comment

Pyrimidine-2-carboxylate (pmc) ligand exhibits a N_2_O_2_ donor set with a charge of -1. Due it is rigidity and directionality, the pmc ligand has been used in the construction of coordination polymers exhibiting permanent microporosity for gas storage (Sava *et al.*, 2008) and antiferromagnetism with *T*_N_ = 21 K (Rodríguez-Diéguez *et al.*, 2007). Here, we report the crystal structure of a novel cadmium(II) coordination polymer containing the pmc ligand, [Cd(C_5_H_3_N_2_O_2_)(H_2_O)(NO_3_)] (I). The pmc ligand was unexpectedly hydrolyzed *in situ* from the 1,4–dihydro–3,6–bis(2'–pyrimidyl)–1,2,4,5–tetrazine (H_2_bmtz) ligand during the crystallization process.

The immediate coordination environment about the cadmium atom in I is shown in Fig. 1 revealing that the Cd(II) atom is heptacoordinate in a distorted capped octahedral geometry constructed by two N and two O atoms from two different pcm ligands, one O atom from a third pcm ligand, and two O atoms of disordered nitrate anions or water molecules. The Cd—N and Cd—O bond distances (Table 1) agree with those found in other N,O–chelate Cd(II) complexes (Sava *et al.*, 2008; Zhang *et al.*, 2008). Each Cd(II) is connected to four other Cd atoms through three pmc ligands generating two dimensional sheets parallel to (201), Fig. 2. Within the sheets, the Cd···Cd distances through the *µ*_2_–carboxylate bridge and the Cd···Cd distances across the pmc ligands are 3.9714 (4) and 6.2427 (3) Å, respectively. The sheets are stabilized by inversion-related pairs of intermolecular O—H···O hydrogen bonds between the coordinated water and nitrate molecules (Table 2). There are, however, no π–π interactions between adjacent pyrimidine rings within the sheets. The distance between *C*_g_ to *C*_g_ of the pyrimidine rings of the pmc ligands is 4.114 (3) Å, which is out the range (3.3–3.8 Å) considered for significant π–π interactions (Janiak, 2000). Further intermolecular O—H···O hydrogen bonds involving the nitrate anions and coordinated water molecules (Table 2) link the sheets into a three dimensional supramolecular network, Fig. 3.

### Experimental

Cadmium nitrate tetrahydrate (30 mg, 0.10 mmol) was dissolved in 2 ml acetonitrile in a glass vial. A solution of 1,4-dihydro-3,6-bis(2'-pyrimidyl)-1,2,4,5-tetrazine (10 mg, 0.04 mmol) in 2 ml dichloromethane was carefully layered on top of the acetonitrile solution. The reaction mixture was allowed to stand undisturbed at room temperature. Pale-green plate-like crystals of I were obtained after three months (yield *ca*. 7% based on Cd source).

### Refinement

The carbon-bound hydrogen atoms were placed in geometrically idealized positions and constrained to ride on their parent atom positions with C—H distances of 0.93 Å and with *U*_iso_(H) = 1.2*U*_eq_(C) for the aromatic H atoms. The hydrogen atoms attached to oxygen atoms of the water molecules were located in a difference Fourier map and refined as riding in their as-found positions with a *DFIX* restraint of O—H distance at 0.900±0.001 Å, with *U*_iso_(H) = 1.2*U*_eq_(O). The nitrate anion was shown to be disordered over two sites in a 0.57 (7) and 0.43 (7) ratio. The N—O bond lengths were restrained to 1.25±0.01 Å and the O···O distances to 2.17±0.01 Å. The highest peak in the final electron density difference map is located 0.85 Å from Cd1 atom.

### Crystal data

**Table:**

[Cd(C_5_H_3_N_2_O_2_)(NO_3_)(H_2_O)]	*Z* = 4
*M**_r_* = 315.52	*F*(000) = 608
Monoclinic, *P*2_1_/*n*	*D*_x_ = 2.398 Mg m^−^^3^
Hall symbol: -P 2yn	Mo *K*α radiation, λ = 0.71073 Å
*a* = 8.1963 (2) Å	µ = 2.52 mm^−^^1^
*b* = 10.1554 (3) Å	*T* = 298 K
*c* = 11.0057 (3) Å	Plate, pale-green
β = 107.435 (3)°	0.23 × 0.20 × 0.14 mm
*V* = 873.99 (4) Å^3^	

### Data collection

**Table:**

Bruker SMART APEX CCD area detector diffractometer	2030 independent reflections
Radiation source: fine-focus sealed tube	1780 reflections with *I* > 2σ(*I*)
Graphite monochromator	*R*_int_ = 0.031
Detector resolution: 8 pixels mm^-1^	θ_max_ = 28.7°, θ_min_ = 2.8°
ω and φ scans	*h* = −10→10
Absorption correction: multi-scan (*SADABS*; Bruker, 2001)	*k* = −13→13
*T*_min_ = 0.596, *T*_max_ = 0.720	*l* = −9→14
5450 measured reflections	

### Refinement

**Table:**

Refinement on *F*^2^	Primary atom site location: structure-invariant direct methods
Least-squares matrix: full	Secondary atom site location: difference Fourier map
*R*[*F*^2^ > 2σ(*F*^2^)] = 0.028	Hydrogen site location: inferred from neighbouring sites
*wR*(*F*^2^) = 0.070	H atoms treated by a mixture of independent and constrained refinement
*S* = 1.04	*w* = 1/[σ^2^(*F*_o_^2^) + (0.0397*P*)^2^ + 0.7721*P*] where *P* = (*F*_o_^2^ + 2*F*_c_^2^)/3
2030 reflections	(Δ/σ)_max_ < 0.001
163 parameters	Δρ_max_ = 1.52 e Å^−^^3^
56 restraints	Δρ_min_ = −0.64 e Å^−^^3^

### Special details

**Table:**

Geometry. All e.s.d.'s (except the e.s.d. in the dihedral angle between two l.s. planes) are estimated using the full covariance matrix. The cell e.s.d.'s are taken into account individually in the estimation of e.s.d.'s in distances, angles and torsion angles; correlations between e.s.d.'s in cell parameters are only used when they are defined by crystal symmetry. An approximate (isotropic) treatment of cell e.s.d.'s is used for estimating e.s.d.'s involving l.s. planes.
Refinement. Refinement of *F*^2^ against all reflections. The weighted *R*-factor *wR* and goodness of fit *S* are based on *F*^2^, conventional *R*-factors *R* are based on *F*, with *F* set to zero for negative *F*^2^. The threshold expression of *F*^2^ > σ(*F*^2^) is used only for calculating *R*-factors(gt) *etc*. and is not relevant to the choice of reflections for refinement. *R*-factors based on *F*^2^ are statistically about twice as large as those based on *F*, and *R*- factors based on ALL data will be even larger.

### Fractional atomic coordinates and isotropic or equivalent isotropic displacement parameters (Å^2^)

**Table:**

	*x*	*y*	*z*	*U*_iso_*/*U*_eq_	Occ. (<1)
C1	0.6403 (5)	0.4196 (4)	0.3921 (3)	0.0363 (8)	
H1	0.5792	0.4287	0.3064	0.044*	
C2	0.7507 (5)	0.5181 (4)	0.4522 (3)	0.0376 (8)	
H2	0.7663	0.5931	0.4085	0.045*	
C3	0.8371 (4)	0.5016 (4)	0.5793 (3)	0.0311 (7)	
H3	0.9123	0.5668	0.6218	0.037*	
C4	0.7102 (4)	0.3012 (3)	0.5773 (3)	0.0224 (6)	
C5	0.6984 (4)	0.1763 (3)	0.6481 (3)	0.0225 (6)	
Cd1	0.42986 (3)	0.13550 (2)	0.364191 (19)	0.02405 (10)	
N1	0.6194 (3)	0.3106 (3)	0.4549 (2)	0.0274 (6)	
N2	0.8160 (3)	0.3947 (3)	0.6429 (2)	0.0255 (6)	
N3	0.0877 (2)	0.1564 (3)	0.4301 (2)	0.0448 (8)	
O1	0.5992 (3)	0.0886 (2)	0.5857 (2)	0.0275 (5)	
O2	0.7882 (3)	0.1688 (2)	0.7600 (2)	0.0346 (6)	
O3	0.6725 (3)	0.0370 (3)	0.3242 (2)	0.0417 (6)	
H3A	0.701 (6)	−0.031 (3)	0.379 (4)	0.073 (17)*	
H3B	0.766 (5)	0.088 (5)	0.356 (6)	0.11 (3)*	
O4	0.2444 (2)	0.1673 (3)	0.4869 (2)	0.0429 (7)	
O5A	−0.0195 (4)	0.192 (4)	0.4840 (14)	0.090 (4)	0.57 (7)
O5B	−0.0159 (7)	0.143 (4)	0.4926 (5)	0.073 (5)	0.43 (7)
O6A	0.0390 (5)	0.116 (3)	0.3175 (9)	0.080 (4)	0.57 (7)
O6B	0.0359 (8)	0.156 (5)	0.3109 (3)	0.092 (7)	0.43 (7)

### Atomic displacement parameters (Å^2^)

**Table:**

	*U*^11^	*U*^22^	*U*^33^	*U*^12^	*U*^13^	*U*^23^
C1	0.0401 (18)	0.040 (2)	0.0222 (16)	−0.0017 (16)	−0.0007 (14)	0.0075 (15)
C2	0.048 (2)	0.032 (2)	0.0294 (17)	−0.0030 (15)	0.0072 (15)	0.0079 (15)
C3	0.0363 (17)	0.0264 (17)	0.0306 (16)	−0.0060 (13)	0.0098 (14)	−0.0011 (14)
C4	0.0221 (13)	0.0235 (16)	0.0191 (14)	0.0011 (11)	0.0023 (11)	−0.0007 (12)
C5	0.0202 (13)	0.0266 (16)	0.0180 (13)	−0.0018 (11)	0.0018 (11)	−0.0024 (12)
Cd1	0.02407 (13)	0.02804 (16)	0.01425 (13)	0.00143 (8)	−0.00306 (8)	−0.00004 (9)
N1	0.0257 (12)	0.0317 (15)	0.0193 (12)	−0.0019 (11)	−0.0017 (10)	0.0007 (11)
N2	0.0265 (13)	0.0273 (14)	0.0186 (12)	−0.0021 (10)	0.0007 (10)	−0.0023 (10)
N3	0.0348 (17)	0.0335 (18)	0.062 (2)	−0.0006 (13)	0.0087 (16)	−0.0008 (16)
O1	0.0282 (11)	0.0268 (12)	0.0208 (10)	−0.0077 (9)	−0.0031 (9)	−0.0001 (9)
O2	0.0408 (14)	0.0335 (13)	0.0181 (11)	−0.0079 (10)	−0.0085 (10)	0.0021 (10)
O3	0.0318 (13)	0.0595 (19)	0.0308 (13)	0.0029 (12)	0.0047 (10)	0.0026 (13)
O4	0.0283 (12)	0.0520 (17)	0.0459 (16)	0.0024 (11)	0.0075 (11)	0.0073 (13)
O5A	0.052 (5)	0.066 (10)	0.172 (9)	0.000 (3)	0.064 (5)	−0.036 (5)
O5B	0.047 (6)	0.059 (12)	0.128 (10)	0.006 (4)	0.049 (6)	−0.024 (5)
O6A	0.113 (9)	0.062 (9)	0.053 (5)	−0.044 (5)	0.005 (5)	−0.006 (3)
O6B	0.088 (9)	0.089 (16)	0.071 (6)	−0.067 (7)	−0.017 (6)	0.000 (6)

### Geometric parameters (Å, º)

**Table:**

C1—N1	1.342 (5)	Cd1—O1^ii^	2.371 (2)
C1—C2	1.378 (5)	Cd1—O2^i^	2.411 (2)
C1—H1	0.9300	Cd1—O3	2.382 (3)
C2—C3	1.375 (5)	Cd1—O4	2.339 (2)
C2—H2	0.9300	N2—Cd1^iii^	2.353 (3)
C3—N2	1.331 (4)	N3—O5A	1.2514 (9)
C3—H3	0.9300	N3—O6B	1.2513 (9)
C4—N1	1.333 (4)	N3—O5B	1.2515 (9)
C4—N2	1.342 (4)	N3—O6A	1.2515 (9)
C4—C5	1.506 (5)	N3—O4	1.2541 (8)
C5—O2	1.233 (4)	O1—Cd1^ii^	2.371 (2)
C5—O1	1.261 (4)	O2—Cd1^iii^	2.411 (2)
Cd1—N1	2.376 (3)	O3—H3A	0.9000 (10)
Cd1—N2^i^	2.353 (3)	O3—H3B	0.9000 (11)
Cd1—O1	2.463 (2)		
			
N1—C1—C2	121.1 (3)	O4—Cd1—O1	74.11 (8)
N1—C1—H1	119.4	N2^i^—Cd1—O1	158.49 (9)
C2—C1—H1	119.4	O1^ii^—Cd1—O1	69.52 (8)
C3—C2—C1	117.7 (3)	N1—Cd1—O1	67.99 (8)
C3—C2—H2	121.2	O3—Cd1—O1	81.24 (9)
C1—C2—H2	121.2	O2^i^—Cd1—O1	132.71 (8)
N2—C3—C2	121.7 (3)	C4—N1—C1	117.5 (3)
N2—C3—H3	119.1	C4—N1—Cd1	117.9 (2)
C2—C3—H3	119.1	C1—N1—Cd1	124.6 (2)
N1—C4—N2	124.6 (3)	C3—N2—C4	117.4 (3)
N1—C4—C5	118.7 (3)	C3—N2—Cd1^iii^	125.3 (2)
N2—C4—C5	116.7 (3)	C4—N2—Cd1^iii^	117.1 (2)
O2—C5—O1	126.5 (3)	O5A—N3—O6B	115.6 (6)
O2—C5—C4	117.1 (3)	O5A—N3—O5B	23.4 (9)
O1—C5—C4	116.3 (3)	O6B—N3—O5B	120.16 (10)
O4—Cd1—N2^i^	119.42 (8)	O5A—N3—O6A	120.16 (10)
O4—Cd1—O1^ii^	82.55 (9)	O6B—N3—O6A	18.7 (19)
N2^i^—Cd1—O1^ii^	94.55 (8)	O5B—N3—O6A	116.1 (5)
O4—Cd1—N1	96.32 (9)	O5A—N3—O4	119.88 (10)
N2^i^—Cd1—N1	122.64 (9)	O6B—N3—O4	119.92 (10)
O1^ii^—Cd1—N1	136.01 (8)	O5B—N3—O4	119.88 (10)
O4—Cd1—O3	152.61 (9)	O6A—N3—O4	119.88 (10)
N2^i^—Cd1—O3	81.25 (9)	C5—O1—Cd1^ii^	130.1 (2)
O1^ii^—Cd1—O3	77.73 (9)	C5—O1—Cd1	118.9 (2)
N1—Cd1—O3	85.12 (10)	Cd1^ii^—O1—Cd1	110.48 (8)
O4—Cd1—O2^i^	81.79 (9)	C5—O2—Cd1^iii^	118.9 (2)
N2^i^—Cd1—O2^i^	68.29 (9)	Cd1—O3—H3A	105 (3)
O1^ii^—Cd1—O2^i^	146.55 (8)	Cd1—O3—H3B	110 (5)
N1—Cd1—O2^i^	75.17 (9)	H3A—O3—H3B	99 (3)
O3—Cd1—O2^i^	124.59 (10)	N3—O4—Cd1	116.46 (16)
			
N1—C1—C2—C3	−0.8 (6)	C5—C4—N2—Cd1^iii^	−9.9 (4)
C1—C2—C3—N2	0.0 (6)	O2—C5—O1—Cd1^ii^	−11.1 (5)
N1—C4—C5—O2	177.6 (3)	C4—C5—O1—Cd1^ii^	167.56 (19)
N2—C4—C5—O2	−0.9 (4)	O2—C5—O1—Cd1	178.3 (3)
N1—C4—C5—O1	−1.2 (4)	C4—C5—O1—Cd1	−3.0 (4)
N2—C4—C5—O1	−179.7 (3)	O4—Cd1—O1—C5	−99.9 (2)
N2—C4—N1—C1	2.4 (5)	N2^i^—Cd1—O1—C5	128.0 (3)
C5—C4—N1—C1	−176.0 (3)	O1^ii^—Cd1—O1—C5	172.3 (3)
N2—C4—N1—Cd1	−176.8 (2)	N1—Cd1—O1—C5	3.9 (2)
C5—C4—N1—Cd1	4.8 (4)	O3—Cd1—O1—C5	92.2 (2)
C2—C1—N1—C4	−0.4 (5)	O2^i^—Cd1—O1—C5	−37.6 (3)
C2—C1—N1—Cd1	178.8 (3)	O4—Cd1—O1—Cd1^ii^	87.84 (11)
O4—Cd1—N1—C4	65.6 (2)	N2^i^—Cd1—O1—Cd1^ii^	−44.3 (3)
N2^i^—Cd1—N1—C4	−163.3 (2)	O1^ii^—Cd1—O1—Cd1^ii^	0.0
O1^ii^—Cd1—N1—C4	−20.2 (3)	N1—Cd1—O1—Cd1^ii^	−168.36 (13)
O3—Cd1—N1—C4	−86.9 (2)	O3—Cd1—O1—Cd1^ii^	−80.10 (11)
O2^i^—Cd1—N1—C4	145.4 (3)	O2^i^—Cd1—O1—Cd1^ii^	150.15 (10)
O1—Cd1—N1—C4	−4.4 (2)	O1—C5—O2—Cd1^iii^	−170.1 (2)
O4—Cd1—N1—C1	−113.5 (3)	C4—C5—O2—Cd1^iii^	11.2 (4)
N2^i^—Cd1—N1—C1	17.6 (3)	O5A—N3—O4—Cd1	−169 (2)
O1^ii^—Cd1—N1—C1	160.7 (3)	O6B—N3—O4—Cd1	−14 (2)
O3—Cd1—N1—C1	94.0 (3)	O5B—N3—O4—Cd1	164 (2)
O2^i^—Cd1—N1—C1	−33.8 (3)	O6A—N3—O4—Cd1	7.7 (15)
O1—Cd1—N1—C1	176.5 (3)	N2^i^—Cd1—O4—N3	5.6 (3)
C2—C3—N2—C4	1.8 (5)	O1^ii^—Cd1—O4—N3	−85.4 (3)
C2—C3—N2—Cd1^iii^	−172.5 (3)	N1—Cd1—O4—N3	138.9 (3)
N1—C4—N2—C3	−3.2 (5)	O3—Cd1—O4—N3	−129.5 (3)
C5—C4—N2—C3	175.3 (3)	O2^i^—Cd1—O4—N3	64.9 (3)
N1—C4—N2—Cd1^iii^	171.6 (2)	O1—Cd1—O4—N3	−156.2 (3)

Symmetry codes: (i) *x*−1/2, −*y*+1/2, *z*−1/2; (ii) −*x*+1, −*y*, −*z*+1; (iii) *x*+1/2, −*y*+1/2, *z*+1/2.

### Hydrogen-bond geometry (Å, º)

**Table:**

*D*—H···*A*	*D*—H	H···*A*	*D*···*A*	*D*—H···*A*
O3—H3*A*···O4^ii^	0.90 (1)	1.98 (1)	2.871 (4)	173 (5)
O3—H3*B*···O5*A*^iv^	0.90 (1)	2.17 (2)	3.045 (14)	164 (6)
O3—H3*B*···O5*B*^iv^	0.90 (1)	2.04 (3)	2.876 (13)	154 (6)

Symmetry codes: (ii) −*x*+1, −*y*, −*z*+1; (iv) *x*+1, *y*, *z*.
